# A framework for protein structure classification and identification of novel protein structures

**DOI:** 10.1186/1471-2105-7-456

**Published:** 2006-10-16

**Authors:** You Jung Kim, Jignesh M Patel

**Affiliations:** 1 Computer Science and Engineering, University of Michigan, 2260 Hayward, Ann Arbor, Ml, USA

## Abstract

**Background:**

Protein structure classification plays a central role in understanding the function of a protein molecule with respect to all known proteins in a structure database. With the rapid increase in the number of new protein structures, the need for *automated *and *accurate *methods for protein classification is increasingly important.

**Results:**

In this paper we present a unified framework for protein structure classification and identification of novel protein structures. The framework consists of a set of components for comparing, classifying, and clustering protein structures. These components allow us to accurately classify proteins into known folds, to detect new protein folds, and to provide a way of clustering the new folds. In our evaluation with SCOP 1.69, our method correctly classifies 86.0%, 87.7%, and 90.5% of new domains at family, superfamily, and fold levels. Furthermore, for protein domains that belong to new domain families, our method is able to produce clusters that closely correspond to the new families in SCOP 1.69. As a result, our method can also be used to suggest new classification groups that contain novel folds.

**Conclusion:**

We have developed a method called proCC for automatically classifying and clustering domains. The method is effective in classifying new domains and suggesting new domain families, and it is also very efficient. A web site offering access to proCC is freely available at

## Background

Classification of protein domains based on their tertiary structure provides a valuable resource that can be used to understand protein function and evolutionary relationships [1]. As a result, several classification databases [1-3] have been developed, of which SCOP [1] and CATH [2] are the most widely used databases. Both databases are hierarchically organized and use protein domains as a basic unit of classification. While SCOP and CATH provide a valuable resource for biologists, these databases are updated only intermittently – for example, over the past three years, SCOP has been updated roughly every six months, and CATH has been updated annually. Updates to these databases require varying degrees of semi-automated methods and manual interpretation. As a result, newly deposited protein structures only show up in the classification hierarchy in the next release cycle of these databases. At the same time, the number of newly determined protein structures has been growing rapidly. For instance, during the past year, more than 5000 structures were deposited in PDB. Also, the number of structures in PDB today is roughly double the number of structures in the year 2000 [4]. This rapid increase in the number of new protein structures makes the need for *automated *classification tools even more important.

We recognize that the manual and semi-automated methods used in SCOP and CATH produce classification hierarchies that are of high quality, and automated methods are unlikely to incorporate the nuanced judgment that an experienced biologist brings to the classification task. Nevertheless, automated methods, if they are highly accurate, can provide a valuable complementary approach in producing classification hierarchies. With the rapid increase in the number of protein structures, automated methods can (and currently do) play an important role as a pre-processing step for producing manually-tuned classification hierarchies.

Recognizing this need, several automatic domain classification methods [5-9] have recently been developed. Superfamily [5] is purely based on sequence comparison criteria. It is efficient, but often fails in correctly classifying remote homologs of structurally similar proteins. Methods such as F2CS [6] and SGM [7] are based purely on structure comparison. They are computationally very efficient and accurate for classifying at the fold level, but not necessarily at the superfamily and family levels. Recent methods [8,9] combine sequence and structure information for classification, and make a classification decision based on a consensus of several sequence and structure comparisons. In general, these methods are more accurate than previous methods, though they are computationally more expensive.

An important issue in automatic protein classification is the ability of a tool to detect new classes (i.e. detecting novel folds). Detecting such new classes is important as novel domain structures are constantly found in newly determined protein structures, and the information about new classes can be effectively used to better understand the new structures and can also be used to assist humans in organizing the new structures into the next version of the classification hierarchy. While many of these existing protein classification methods are very good at classifying new domains into *existing *classes, the effectiveness of these methods in detecting *new *classes is modest. Of the existing classification tools, both SGM [7] and SCOPmap [8] can be used to detect new classes, and as we show in this paper our method is much more effective compared to these two methods for new class detection.

In this paper, we present *proCC *– an automatic, accurate, and efficient classification framework, which consists of three components. Given an unclassified query domain, a structure comparison component employs an index-based method to quickly find domains with similar structures. Then, based on these results, a classification component assigns the query to an existing class label, or marks the query as *unclassified *to indicate that the query domain is potentially a new fold. Finally, a clustering component takes all domains marked as unclassified and runs a clustering method to detect potentially novel folds.

Collectively, these components provide a unified and automated protein domain classification tool. To demonstrate the capabilities of our methods, we have tested our method to predict the classification for new domains in SCOP 1.69 based on prior knowledge of the previous version of SCOP (version 1.67). Our experimental results show that the precision of our method is 86.0%, 87.7%, and 90.5% at the family, superfamily, and fold levels. We also compare our method with SGM and SCOPmap, and show that our methods are about 15–19% more accurate than SGM and comparable to SCOPmap. However, SCOPmap only classifies at the superfamily and the fold levels, whereas our tool also provides a classification at the the family level. More significantly for new fold detection, the predications made by proCC is 20% better than SCOPmap. Our experimental evaluation also shows that our method produces clusters which closely correspond to the new families in SCOP 1.69.

## Results

### Experimental setup and datasets

In this section, we present results measuring the effectiveness of our classification methods. For the empirical evaluation, we employed the experimental strategies used in previous studies [8,9]: namely, domains in an older version of SCOP are used as the set of database domains with known class labels, and domains in a newer version of SCOP are used as the query set. Classification accuracy is measured by comparing the predicted labels with the (known) labels in the newer version of SCOP. In our experiments, SCOP 1.67 and SCOP 1.69 are used as the database and the query set respectively.

SCOP 1.67 and 1.69 contain 65122 and 70859 domains, which are grouped into 2630 and 2845 families respectively. However, in our evaluation theoretical domains and domains with less than 3 SSEs are excluded. After these exclusions, we end up with 58456 and 63745 domains in SCOP 1.67 and 1.69 respectively. Our database is the set of 58456 domains in SCOP 1.67, and our query set is the 5289 newly added domains in SCOP 1.69. We used the ASTRAL Compendium [10] for the PDB-style coordinate information for these SCOP domains. In addition, we used the STRIDE program [11] to generate secondary structure assignments for each domain.

Our implementation is written in C++, and uses the LEDA 3.2R package for the maximum bipartite graph matching, and the *SVM*^*light*^[12] package. The SVM model was trained using SCOP 1.65 and SCOP 1.67 (see the structure classification section in Methods). We used a radial basis function as the kernel with a weight cost set to the ratio of the number of negative examples to the number of positive examples. All experiments were run on a 2.2 GHz Opteron machine, with 4 GB of RAM, and running the Linux 2.6.9 kernel. Throughout this section, we will use the term *class *to refer to a class in the classification scheme.

### Experimental evaluation

#### Precision and computational cost

To measure the effectiveness of our classification method, we compare the predicted classification label (at the fold, superfamily, and family levels) with the actual label in SCOP 1.69 using the following metrics:

Overall precision = (CC + UN)/(TE + TN)

Classification error ratio = CI/(CC + CI)

New class detection ratio = UN/TN

In the above equations, CC is the number of correctly classified domains, and CI is the number of incorrectly classified domains. UN represents the number of domains of new structures which are not in existing classes and therefore are correctly marked as *unclassified*. UE is the number of domains which should have been classified into existing classes, but which are marked as *unclassified *by our method. (Note *CC *+ *CI *is the total number of domains that are assigned some labels by our method, and *UN *+ *UE *is the total number of domains that are tagged as *unclassified *by our method.) TE represents the total number of domains in common classes in SCOP 1.67 and SCOP 1.69, and TN represents the total number of domains in new classes in SCOP 1.69.

*Overall precision *measures how many proteins are correctly classified or correctly labeled as unclassified. The *classification error ratio *measures how many errors are made when query domains are assigned actual labels. *A new class detection ratio *measures how effectively a method can detect domains that are in new classification classes.

The results for this experiment are shown in Table 1. As can be seen from this table, our classification method is highly accurate and is fairly effective in detecting domains that are in new classification classes. With respect to the computation time for classification, the computation cost is linearly proportional to the number of SSE triplets in the query. The average number of SSEs per domain is about 77, and for queries of this size, our method requires about 30 seconds of execution time. Of this computation time, the index matching component takes about 38% of the time (This index search time is about 8 times faster than a full scan of the file that has all the SSE triplets). About 56% of the computation time is spent on the overall structure matching component (the bipartite graph matching method), and the remaining 6% of the time is spent for program setup, input and output processing, and SVM classification (see the Methods section for description of these components).

**Table 1:** Classification result for proCC using new domains in SCOP 1.69

	**Classified domains**		**Unclassified domains**		**Total domains**		**Overall precision**	**Classification error**	**New class detection ratio**
	Correct CC	Incorrect CI	New classes UN	Existing classes UE	New classes TN	Existing classes TE	(CC+UN)(TN+TE) MathType@MTEF@5@5@+=feaafiart1ev1aaatCvAUfKttLearuWrP9MDH5MBPbIqV92AaeXatLxBI9gBaebbnrfifHhDYfgasaacH8akY=wiFfYdH8Gipec8Eeeu0xXdbba9frFj0=OqFfea0dXdd9vqai=hGuQ8kuc9pgc9s8qqaq=dirpe0xb9q8qiLsFr0=vr0=vr0dc8meaabaqaciaacaGaaeqabaqabeGadaaakeaadaWcaaqaaiabcIcaOiabboeadjabboeadjabgUcaRiabbwfavjabb6eaojabcMcaPaqaaiabcIcaOiabbsfaujabb6eaojabgUcaRiabbsfaujabbweafjabcMcaPaaaaaa@3AE4@	CI(CC+CI) MathType@MTEF@5@5@+=feaafiart1ev1aaatCvAUfKttLearuWrP9MDH5MBPbIqV92AaeXatLxBI9gBaebbnrfifHhDYfgasaacH8akY=wiFfYdH8Gipec8Eeeu0xXdbba9frFj0=OqFfea0dXdd9vqai=hGuQ8kuc9pgc9s8qqaq=dirpe0xb9q8qiLsFr0=vr0=vr0dc8meaabaqaciaacaGaaeqabaqabeGadaaakeaadaWcaaqaaiabboeadjabbMeajbqaaiabcIcaOiabboeadjabboeadjabgUcaRiabboeadjabbMeajjabcMcaPaaaaaa@35B6@	UNTN MathType@MTEF@5@5@+=feaafiart1ev1aaatCvAUfKttLearuWrP9MDH5MBPbIqV92AaeXatLxBI9gBaebbnrfifHhDYfgasaacH8akY=wiFfYdH8Gipec8Eeeu0xXdbba9frFj0=OqFfea0dXdd9vqai=hGuQ8kuc9pgc9s8qqaq=dirpe0xb9q8qiLsFr0=vr0=vr0dc8meaabaqaciaacaGaaeqabaqabeGadaaakeaadaWcaaqaaiabbwfavjabb6eaobqaaiabbsfaujabb6eaobaaaaa@3162@
Family	4008	347	555	379	726	4563	86.3%	8.0%	76.5%
Superfamily	4321	154	292	522	353	4936	87.2%	3.4%	82.7%
Fold	4597	159	153	380	209	5080	90.1%	3.3%	75.0%

This table shows the result of classifying 5298 new domains in SCOP 1.69 using proCC.

#### Comparison with other methods

A number of methods have previously been proposed for automatic classification [5,7-9]. In evaluating performance, we considered comparing our method with each of these methods. However, some of these methods are not suitable for comparison because of the following reasons. Currently, a fair comparison with Superfamily [5] is not possible since a SCOP 1.67 Hidden Markov Model is required for comparison, and this model is currently not available (Personal Communication, Derek Wilson, 2006). Comparison with [9] is not possible since its implementation or result data sets are not available.

Therefore, in this section, we compare our method with SGM [7] and SCOPmap [8]. The SGM method is a classification method based on 30-dimensional Gaussian integrals of protein structures, and nearest neighbor classification. The SGM method has been shown to be very fast and effective for classifying CATH. SCOPmap is a consensus-based method that uses seven different sequence and structure comparison methods. SCOPmap has been extensively compared with Superfamily, and has been shown to be more accurate than Superfamily [8].

##### Comparison with SGM

Before presenting the results with SGM, we note that the performance of SGM can change depending on adjustable parameters, such as the distance ratio cutoff in SGM. We experimented with a variety of parameter settings for SGM and found that settings that increase the new class detection ratio (or decrease the classification error ratio), degrade the overall precision. To select a reasonable baseline for comparison, we picked parameter values for SGM which produce a new class detection ratio similar to our method. With this method, we end up with distance ratio cutoff values of 1.22, 1.23, and 1.23 at the family, superfamily, and fold levels respectively.

The results comparing SGM and proCC for the 5289 new domains in SCOP 1.69 are shown in Table 2. Although SGM was very effective for classifying CATH, this method is less successful with SCOP. As these result shows, our method is 15–19% more accurate than SGM at the family, superfamily, and fold levels, and makes fewer misclassification mistakes.

**Table 2:** The comparison between SGM and proCC

	**Overall precision**		**Classification error ratio**		**New class detection ratio**	
	SGM	proCC	SGM	proCC	SGM	proCC
Family	71.3%	86.3%	19.7%	8.0%	77.4%	76.5%
Superfamily	69.6%	87.2%	17.0%	3.4%	82.2%	82.7%
Fold	71.3%	90.1%	15.7%	3.3%	76.6%	75.0%

This table shows the result of comparing SGM and proCC for classifying 5298 new domains in SCOP 1.69.

We have also evaluated proCC, and compared it with SGM, using CATH (SGM was originally only tested against CATH). We used CATH 2.0 and CATH 2.4 as the database and query domains. The overall precision of the SGM method in classifying CATH is 93.9%, 94.5%, 94.7%, and 97.1% at the H, T, A, and C levels, whereas the overall precision of our method is 94.1%, 95.6%, 95.6% and 97.2% at the H, T, A, and C levels. Compared to SCOP, both methods generate more accurate results with CATH. However, the higher precision with CATH is expected since CATH uses a broader definition of fold, i.e. there are fewer folds in the CATH classification compared to SCOP [13].

In addition, we have also compared the sensitivity and specificity of proCC with SGM and plotted standard ROC curves. These results are presented in the Additional file 1.

##### Comparison with SCOPmap

In this section, we present results comparing SCOPmap and our proCC method. In comparison with SCOPmap, we note that SCOPmap takes as input a query protein chain, identifies domains by aligning the query protein chain to sequences and structures in its database, and assigns a classification label to each identified domain. On the other hand, the input to proCC is a domain rather than a protein chain. So for comparison with SCOPmap, we first ran a domain prediction method with query protein chains to identify the domain boundaries. Then, we ran our classification method on the identified domains. For domain boundary prediction, we used the SSEP-domain method [14], which was shown to be very accurate in the CAFASP4-DP competition [15].

We compared SCOPmap and proCC using 2773 new single domain chains in SCOP 1.69. For this experiment, multi-domain chains are excluded, due to the difficulty in measuring effectiveness objectively (In the case of multi-domain chains, the number of predicted domains, predicted domain boundaries, and the number of correct domain classification assignments all need to be considered, and there is no systematic way of differentiating these effects from the actual classification effectiveness which we aim to evaluate).

Initially, we attempted to run SCOPmap on the 2773 chains. However, running SCOPmap on these 2773 chains takes an enormous amount of computational resource requiring approximately 2–3 hours to process each individual chain (Personal Communication, Sara Cheek, 2006). Due to this high computation cost, new proteins are typically classified using large clusters and classification results are posted at . Therefore, we compared our method with SCOPmap based on the latest result posted on the SCOPmap ftp site.

Finally, while our method can predict the family, superfamily, and fold labels, SCOPmap primarily predicts the superfamily label, and only predicts the fold label for queries that it cannot assign a superfamily label. SCOPmap never predicts a family label. Since the main classification prediction made by SCOPmap is at the superfamily level, for this evaluation we compared the classification effectiveness only at this level. The results of this evaluation are shown in Table 3. From this table we can make the following observations:

**Table 3:** The comparison between SCOPmap and proCC using the predicted Superfamily SCOP labels

	**Classified with correct domain boundary**		**Unclassified with correct domain boundary**		**Incorrect domain boundary**	**Overall precision**	**New class detection ratio**	**Estimated average execution time**
	Correct CC	Incorrect CI	New classes UN	Existing classes UE	ID	(CC+UN)2773 MathType@MTEF@5@5@+=feaafiart1ev1aaatCvAUfKttLearuWrP9MDH5MBPbIqV92AaeXatLxBI9gBaebbnrfifHhDYfgasaacH8akY=wiFfYdH8Gipec8Eeeu0xXdbba9frFj0=OqFfea0dXdd9vqai=hGuQ8kuc9pgc9s8qqaq=dirpe0xb9q8qiLsFr0=vr0=vr0dc8meaabaqaciaacaGaaeqabaqabeGadaaakeaadaWcaaqaaiabbIcaOiabboeadHqaaiab=neadjab=TcaRiab=vfavjab=5eaojabbMcaPaqaaiabbkdaYiabbEda3iabbEda3iabbodaZaaaaaa@377A@	UN307 MathType@MTEF@5@5@+=feaafiart1ev1aaatCvAUfKttLearuWrP9MDH5MBPbIqV92AaeXatLxBI9gBaebbnrfifHhDYfgasaacH8akY=wiFfYdH8Gipec8Eeeu0xXdbba9frFj0=OqFfea0dXdd9vqai=hGuQ8kuc9pgc9s8qqaq=dirpe0xb9q8qiLsFr0=vr0=vr0dc8meaabaqaciaacaGaaeqabaqabeGadaaakeaadaWcaaqaaiabbwfavjabb6eaobqaaiabbodaZiabbcdaWiabbEda3aaaaaa@31D9@	
SCOPmap	2069	65	190	212	237	81.5%	61.9%	2–3 hours per query
proCC	2025	75	246	275	152	81.9%	80.1%	9 minutes per query

This table shows the result of classifying 2773 single domain chains in SCOP 1.69. All numbers reported in column 2–6 are in terms of the number of chains (or domains due to the fact that we used single domain chains). Column 2–3 show the number of single domain chains which are correctly identified as single domain chains and are classified to known superfamilies. Column 4–5 show the number of single domain chains which are correctly identified as single domain chains and are labeled as unclassified. Column 6 shows the number of single chain domains which are incorrectly identified as multi-domain chains.

##### (1) Overall precision

By examining column 5 in Table 3, we observe that the overall precision of SCOPmap is marginally lower than proCC with the SSEP-domain prediction method. From column 4, we also observe that the SSEP-domain prediction method performs better than SCOPmap in identifying single domain chains. To isolate the effect of domain prediction from the classification accuracy, we also measured overall precision as (CC + CI)/(2773 – ID). This adjusted overall precision is 89.1% and 86.7% for SCOPmap and proCC respectively. We note that SCOPmap is tightly coupled with its domain prediction method, and considered as an entire package, proCC coupled with SSEP provides slightly higher overall precision than SCOPmap. Furthermore, the added advantage of our approach is that it can be coupled with any domain prediction method allowing our approach to easily leverage future improvements in domain predication methods.

##### (2) Detection of novel structures

From column 6 in Table 3, we observe that with respect to detecting novel structures, our method is about 20% more accurate than SCOPmap. The reason for this difference is that SCOPmap aggressively classifies a query into a known classification class if at least one of the 7 sequence and structure comparison methods can find a significant match to the query. This approach can be effective when the query belongs to a known class, but is vulnerable to making false predications for queries that have novel structures, especially when classification boundaries for those structures are ambiguous. On the other hand, our method makes a classification decision based on a sophisticated decision model, which distinguishes novel protein structures from known protein structures based on knowledge learned from a prior classification database.

##### (3) Computational cost

With respect to computation time (see the last column in Table 3), our method has a clear advantage over SCOPmap. While SCOPmap takes on average 2–3 hours per query, our method can classify a query on average in 9 minutes. Of these 9 minutes, on average 8 minutes are spent on the SSEP domain prediction web service, and on average only 1 minute is spent in our classification method. We recognize that a technique to address the significantly higher computational cost of SCOPmap is to employ a large cluster. While this solution is practical in some cases (although very costly), with the increasing rate of production of new structures it may be more practical to employ a much cheaper solution like proCC which has comparable precision and offers more flexibility as it can be coupled with any domain prediction tool. Finally, we note that in contrast of SCOPmap, proCC also provides classification predictions for the SCOP family level. Such predictions are useful as it is known that several domains in the same superfamily can be functionally divergent, and a more fine-grained family level classification is more useful for predicting domain functions [16].

#### Detection and clustering of novel families, superfamilies, and folds

From the query set of 5289 domains, our classification method labels 934 domains as *unclassified*. As a way of identifying and describing novel families, superfamilies, and folds among these unclassified domains, we ran the MCL clustering algorithm on a graph constructed using these unclassified domains. To construct a graph for clustering, a threshold value for structure similarity is required (see the identification and clustering of novel structures section in Methods). In addition, for the clustering at the different SCOP levels, different threshold values are needed. For this experiment, we set the threshold value to 0.4, 0.32, and 0.3 for the family, superfamily, and fold levels respectively, based on the observation that more than 90% of correctly classified proteins have a similarity score above these values with their nearest structure neighbor in the same SCOP family, superfamily, and fold.

To measure the capability of the automated method in identifying novel SCOP families, we compared the automatically produced clusters with the family level classes in SCOP 1.69. The 934 unclassified domains are spread across 320 families in SCOP 1.69. For these domains, the automated method produced 358 clusters. To check the agreement between SCOP and the automatically generated clusters, we generated class labels for the clusters based on the most common family label in a cluster. Based on this class label assignment, each SCOP family is paired with one or zero cluster having the same class label. When more than one cluster maps to the same SCOP family, we count only the assignment of one of the automatically generated cluster; this cluster is the one in which the number of domains that correctly match the SCOP family label is highest amongst the set of clusters that also have the same SCOP family label. We then counted the number of common clusters/families that were "correctly" mapped, and found that there are 301 common clusters between the two classifications. Then, for each correctly mapped cluster, we counted the number of actual domains in the cluster that had the same label as the corresponding SCOP family. This total is 822, which is 88% of the total number of unclassified domains.

Using the same method, we also computed the clustering effectiveness at the superfamily and fold levels. These results are shown in Table 4.

**Table 4:** The clustering effectiveness at the SCOP family, superfamily, and fold levels

	**SCOP Classes (A)**	**MCL Clusters (B)**	**# of common clusters/classes (C)**	**# of correctly labeled domains in (C)**
Family	320	358	301	822 (88%)
Superfamily	260	327	234	731 (78%)
Fold	200	318	191	670 (72%)

This table shows the result of clustering 934 unclassified domains at the SCOP family, superfamily, and fold levels. Column 2 shows the number of SCOP families, superfamilies, and folds that these 934 domains are spread across. Column 3 shows the number of automatically generated clusters at each SCOP level. Column 4 shows the number of common clusters/SCOP classes that were correctly mapped. Column 5 shows the number of actual domains in the cluster that had the same label as the corresponding SCOP class.

In Table 4, of the 358 identified clusters at the family level, 159 clusters actually correspond to 159 novel families in SCOP 1.69, which is 74% of the 215 total number of novel families introduced in SCOP 1.69. At the superfamily level, out of 327 identified clusters, 62 clusters actually correspond to 62 novel superfamilies in SCOP 1.69, which is 65% of the 95 total number of novel superfamilies introduced in SCOP 1.69. At the fold level, out of 318 identified clusters, 46 clusters actually correspond to 46 novel families in SCOP 1.69, which is 75% of the 61 total number of novel families introduced in SCOP 1.69. In addition, to measure the extent of homogeneity in automatically generated clusters, we also evaluated the quality of clusters using a measure called "cluster purity" [17]. It is 1 when all domains in the same cluster have perfect agreement in their class labels, and it is defined as:

Cluster  Purity(ℂ,S)=1N∑c∈ℂmax⁡S∈S|C∩S|
 MathType@MTEF@5@5@+=feaafiart1ev1aaatCvAUfKttLearuWrP9MDH5MBPbIqV92AaeXatLxBI9gBaebbnrfifHhDYfgasaacH8akY=wiFfYdH8Gipec8Eeeu0xXdbba9frFj0=OqFfea0dXdd9vqai=hGuQ8kuc9pgc9s8qqaq=dirpe0xb9q8qiLsFr0=vr0=vr0dc8meaabaqaciaacaGaaeqabaqabeGadaaakeaacqWGdbWqcqWGSbaBcqWG1bqDcqWGZbWCcqWG0baDcqWGLbqzcqWGYbGCcaaMc8UaaGPaVlabdcfaqjabdwha1jabdkhaYjabdMgaPjabdsha0jabdMha5jabcIcaOmrr1ngBPrwtHrhAYaqeguuDJXwAKbstHrhAGq1DVbaceaGae8NaHmecbaGae4hlaWIae8NKWpLae4xkaKcccaGae0xpa0ZaaSaaaeaacqqFXaqmaeaaieGacqaFobGtaaWaaabeaeaacyGGTbqBcqGGHbqycqGG4baEdaWgaaWcbaGaem4uamLaeyicI4Sae8NKWpfabeaakiabcYha8jabdoeadjabgMIihdWcbaGaem4yamMaeyicI4Sae8NaHmeabeqdcqGHris5aOGaem4uamLaeiiFaWhaaa@6A24@

In the above equation, *C *is a cluster in the set of MCL clusters ℂ
 MathType@MTEF@5@5@+=feaafiart1ev1aaatCvAUfKttLearuWrP9MDH5MBPbIqV92AaeXatLxBI9gBaebbnrfifHhDYfgasaacH8akY=wiFfYdH8Gipec8Eeeu0xXdbba9frFj0=OqFfea0dXdd9vqai=hGuQ8kuc9pgc9s8qqaq=dirpe0xb9q8qiLsFr0=vr0=vr0dc8meaabaqaciaacaGaaeqabaqabeGadaaakeaatuuDJXwAK1uy0HMmaeHbfv3ySLgzG0uy0HgiuD3BaGabaiab=jqidbaa@3739@, *S *is a family in the set of SCOP families S
 MathType@MTEF@5@5@+=feaafiart1ev1aaatCvAUfKttLearuWrP9MDH5MBPbIqV92AaeXatLxBI9gBaebbnrfifHhDYfgasaacH8akY=wiFfYdH8Gipec8Eeeu0xXdbba9frFj0=OqFfea0dXdd9vqai=hGuQ8kuc9pgc9s8qqaq=dirpe0xb9q8qiLsFr0=vr0=vr0dc8meaabaqaciaacaGaaeqabaqabeGadaaakeaatuuDJXwAK1uy0HMmaeHbfv3ySLgzG0uy0HgiuD3BaGabaiab=jj8tbaa@38A8@, and *N *is the total number of domains in S
 MathType@MTEF@5@5@+=feaafiart1ev1aaatCvAUfKttLearuWrP9MDH5MBPbIqV92AaeXatLxBI9gBaebbnrfifHhDYfgasaacH8akY=wiFfYdH8Gipec8Eeeu0xXdbba9frFj0=OqFfea0dXdd9vqai=hGuQ8kuc9pgc9s8qqaq=dirpe0xb9q8qiLsFr0=vr0=vr0dc8meaabaqaciaacaGaaeqabaqabeGadaaakeaatuuDJXwAK1uy0HMmaeHbfv3ySLgzG0uy0HgiuD3BaGabaiab=jj8tbaa@38A8@.

**Figure 1:**
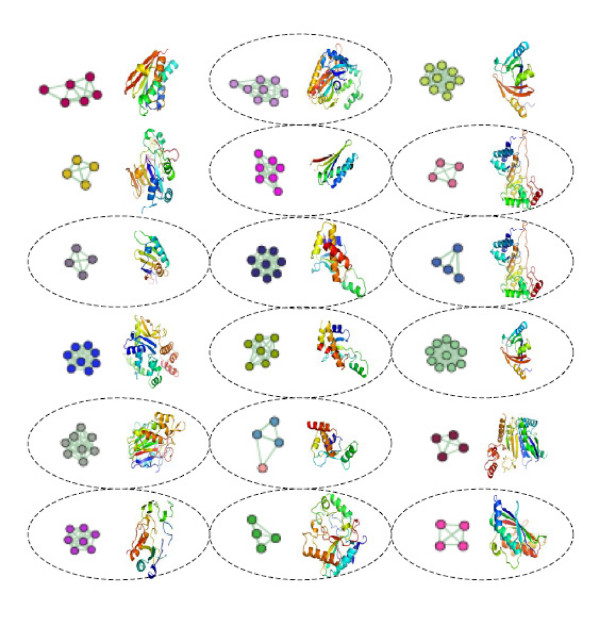
**Assessing the quality of the automatically generated clusters**. This figure shows the automatically generated family-level clusters for the unclassified domains in the SCOP 1.69 "d" class (i.e. the alpha and beta proteins (a+b)). This figure also shows the representative domain structures for each cluster. A connected graph corresponds to an automatically detected MCL cluster. The ellipses indicate the novel families in SCOP 1.69. The MCL clusters are assigned a family-level label based on the most common family-level label in the cluster. Within a cluster, the nodes with the same color indicate that all these nodes have the same family-level label. To keep this figure simple, only clusters with more than four domains are shown. There are an additional of 79 clusters that matched the SCOP family label, and of these 30 clusters correspond to new families in SCOP 1.69. This figure was generated using BioLayout [31] and PyMol [32].

Using this measure, the cluster purity of the MCL clusters is 0.96, 0.95, and 0.96 at the SCOP family, superfamily, and fold levels respectively. This high cluster purity value shows that our clustering method produces clusters that have a high degree of agreement with the SCOP classes. An example of automatically clustered novel SCOP families is shown in Figure 1.

## Discussion

### Applications for efficient structure comparison

In general, existing protein classification methods have focused on classifying new domains into existing classification hierarchies. However, it has been observed that in SCOP previously classified domains are often rearranged in subsequent releases, as new structures sometimes reveal more relationship amongst new and existing domains [18]. Therefore, in addition to classifying new structures, it is to automatically detect such potential rearrangements.

One way of approaching this problem is to perform an all-to-all comparison with existing and new domains, and then generate clusters using a clustering method. For instance, if the introduction of a new domain provides evidence connecting previously unrelated domains, a cluster that consist of these domains can be found, suggesting some potential rearrangements involving these domains.

In performing this task, along with clustering techniques, an efficient and accurate structure comparison method is crucial since one has to compare each pair of structures (*O*(*n*^2^) comparisons). Our structure comparison method (see structure comparison section in Methods), is very efficient and could be a suitable choice for this task.

Incorporating this functionality to continually detect rearrangements of the classification hierarchy into our classification framework will be part of our future work.

### Integration with domain prediction methods

To make a domain classification truly automatic, given a protein structure, first the domain boundaries must be identified. The domain boundary prediction problem is well recognized as a crucial component for functional classification and structure prediction [14], and there are a number of competing domain prediction methods [19]. The proCC method provides a framework which allows us to couple our classification method with any domain prediction tool. While we have used the SSEP-domain method in our current study, other domain prediction methods, for instance, Rosseta-Ginzu [20], which is more accurate but slower, can be used to potentially produce even better classification results. In addition, the loose coupling between the classification and domain prediction components will easily allow us to leverage future advances that are likely to be made in domain boundary prediction methods.

## Conclusion

In this paper we have described a method called proCC for automatically classifying proteins. Using extensive experimental evaluation, we have demonstrated that our method often has higher accuracy compared to existing automated methods. Our method is also very effective in predicting new folds, and is very efficient. While our method cannot completely remove the need for manual intervention that is invariably needed in producing high-quality classification hierarchies such as SCOP and CATH, it can provide a valuable complimentary method for classifying new domains that have not been incorporated into the latest releases of these databases. In addition, our method can also help the curators of these databases in reorganizing the existing classification hierarchies to accommodate new protein structures.

## Methods

Our proCC method consists of a pipeline of the following three modules: structure comparison, structure classification, and clustering. Given a new query protein domain, the structure comparison module finds the top *k *structures that are similar to the query. Then, based on these results, the classification of the query domain is performed using the class label information from the *k *nearest structural neighbors, and a support vector machine (SVM) [21]. This second step may label a query domain as *unclassified *if the classification module cannot assign a class label with enough confidence. Finally, for the domains that are labeled as unclassified by the previous step, a clustering module identifies cluster boundaries as a way of suggesting groups of domains that are potentially in novel folds. Each of these three steps is described in more detail below.

### Structure comparison

The protein domain structure comparison module employs an index structure to rapidly find structures that are similar to the query. The basic unit for comparing structure similarity is a triplet of secondary structure elements (SSEs). Comparing protein structures using SSEs has been used before [3,22,23] as it is more efficient for computing structure similarity, compared to using the actual atomic coordinates of the *C*_*α *_atoms, as is done in DALI [24] and CE [25]. In addition, since domains are classified according to the composition and the spatial arrangement of SSEs in common classification databases such as SCOP and CATH, structure comparison based on SSEs is more natural for the purpose of structure classification. To find the top *k *domains that are similar to a given domain, the following steps are performed in order.

1. Each domain in the database is decomposed into a set of SSE triplets. A 10-dimensional vector is used to represent the SSE triplet, and an index is constructed over all the SSE triplets in the database. The query protein domain is also decomposed into SSE triplets.

2. For each SSE triplet in the query, matching SSE triplets are retrieved using the index. Based on the hits from this index probe, a similarity score between the matching triplets is calculated.

3. For each target domain in the database, a weighted bipartite graph is generated based on the SSE triplet matching results. For each target graph, a maximum weighted bipartite graph matching algorithm is run to compute an overall similarity score between the query and the target. Finally, the top *k *scoring targets are returned as the result of the search.

Each of these three steps is described in detail in the following three subsections.

We note that our method finds all the domains in the database that have at least one or more SSE triplet matches to the query, and *k *is the number of such domains in the database. Therefore, the value for *k *varies depending on the query structure and is automatically determined by our method.

#### Structure representation and indexing

We model each protein domain as a set of SSEs, and represent each SSE using its associated type, length, and a direction vector. Given a SSE *S*_*i*_, its type, denoted as *T*_*i*_, is either an *α *helix or a *β *strand. For a concise representation, loops and turns are excluded. The length of the SSE, denoted as *L*_*i*_, is the number of residues contributing to the formation of that SSE. The direction vector, denoted as *V*_*i*_, is a unit vector, *V*_*i *_= (*X*_*s*_- *X*_*e*_)/||*X*_*s*_- *X*_*e*_||, where *X*_*s *_and *X*_*e *_represent the two end points of the SSE. *X*_*s *_and *X*_*e *_are calculated using the following equations defined in [26].

For an *α *helix, *X*_*s *_and *X*_*e *_are calculated as:

*X*_*s *_= (0.74*X*_*i *_+ *X*_*i*+1 _+ *X*_*i*+2 _+ 0.74*X*_*i*+3_)/3.48

*X*_*e *_= (0.74*X*_*j *_+ *X*_*j*-1 _+ *X*_*j*-2 _+ 0.74*X*_*j*___3_)/3.48

where *X*_*i *_and *X*_*j *_represent the beginning and ending residues of the SSE.

For a *β *strand, *X*_*s *_and *X*_*e *_are calculated as:

*X*_*s *_= (*X*_*i *_+ *X*_*i*+1_)/2

*X*_*e *_= (*X*_*j *_+ *X*_*j*-1_)/2

Since we are interested in indexing SSE triplets, we use the above representation of a single SSE to develop a representation for an SSE triplet. Given three SSEs, *S*_*i*_, *S*_*j *_and *S*_*k*_, the triplet containing these three SSE contains the following information:

• SSE types: *T*_*i*_, *T*_*j*_, *T*_*k*_.

• SSE lengths: *L*_*i*_, *L*_*j*_, *L*_*k*_.

• Angles between each pair of SSEs: *θ*_*ij*_, *θ*_*ik*_, *θ*_*jk *_where *θ*_*ij *_is the angle formed by *S*_*i *_and *S*_*j *_and it is calculated as: cos^-1^(*V*_*i*_·*V*_*j*_) mod 180. (Note that the mod 180 component of the equation is used to allow for similarity matching under coordinate inversion.)

• Distances between each pair of SSEs: *D*_*ij*_, *D*_*ik*_, *D*_*jk *_where *D*_*ij *_is the average of the minimum distances between residues in *S*_*i *_and *S*_*j*_. To calculate *D*_*ij *_the smaller SSE (between *S*_*i *_and *S*_*j*_) is selected. Then, the minimum distances from every residue in *S*_*i *_to every residue in *S*_*j *_are calculated (*S*_*i *_≤ *S*_*j*_) and the average of these minimum distances is used as the SSE distance *D*_*ij*_. Intuitively, this measure aims to concisely capture the distance between two SSEs. The index search (described below) will use these distances to remove pairs of SSEs that have very different inter-SSE distances.

The information describing an SSE triplet is encoded into a compact 10-dimensional vector, which serves as the actual representation of the SSE triplet in an index. This 10-dimensional vector is:

(*TC*, *X*_*i*_, *Y*_*i*_, *X*_*j*_, *Y*_*j*_, *X*_*k*_, *Y*_*k*_, *D*_*jk*_, *D*_*ik*_, *D*_*ij*_)

In this vector representation, amongst the three SSEs, the *i*^*th *^SSE is closest to the N-terminal, and the *k*^*th *^SSE is closest to the C-terminal. *TC *is a three bit value that encodes the types of the three SSEs. The next six values, *X*_*i*_, *Y*_*i*_, *X*_*j*_, *Y*_*j*_, *X*_*k *_and *Y*_*k *_represent the lengths and angles of *S*_*i*_, *S*_*j *_and *S*_*k*_. Each SSE, for instance *S*_*i *_is mapped to a point (*X*_*i*_, *Y*_*i*_) in a 2D Euclidean space where *X*_*i *_= *L*_*i*_*cosθ*_*jk *_and *Y*_*i *_= *L*_*i*_*sinθ*_*jk*_. This transformation to a 2-D Euclidean coordinate allows us to use a conventional spatial index for efficiently locating close neighbors. The last three values, *D*_*jk *_, *D*_*ik*_, and *D*_*ij*_, are the pairwise SSE distance values as defined before.

The 10-dimensional vector representation serves as the key for indexing the SSE triplets. For a given protein domain, rather than inserting an index entry for every SSE triplet, we only insert SSE triplets that have *all *inter-SSE distances less than 20 Å. This cutoff value is based on a similar cutoff that is used in DALI [24]. For the indexing structure, we use the popular R*-tree [27].

In our method, a SSE triplet is used as a basic search unit. While different cardinality for SSE can also be considered (for example a quadruplet or a pair instead of a triplet), the SSE cardinality directly affects the efficiency and the sensitivity of the searches. Using a SSE pair increases the sensitivity of searches, but degrades the search efficiency, especially when searching a large database of domains. Using a SSE quadruplet is more efficient, but may be too conservative in detecting distantly related structures, such as domains in the superfamily or fold levels. We use a SSE triplet to strike a balance between sensitivity and search efficiency. We also note that the use of SSE triplet has been made for similar reasons in previous works [28].

#### SSE triplet matching and index probing

To match a query against a database of proteins, we first decompose the query into all SSE triplets with inter-SSE distances less than 20 Å. We then probe the index with each query triplet and retrieve *target *triplets in the database that are "similar" to the query triplet. Similarity between a query triplet and a target triplet is defined using the scoring model described below.

##### SSE triplet similarity scoring

Given two matching triplets, *T*^*q *^in a query and *T*^*t *^in a target (database), the SSE triplet similarity, denoted by *SC*_*triplet*_(*T*^*q*^, *T*^*t*^) is computed using the following equation.

SCtriplet(Tq,Tt)=SCpair(Sijq,Sijt)+SCpair(Sjkq,Sjkt)+SCpair(Sikq,Sikt)
 MathType@MTEF@5@5@+=feaafiart1ev1aaatCvAUfKttLearuWrP9MDH5MBPbIqV92AaeXatLxBI9gBaebbnrfifHhDYfgasaacH8akY=wiFfYdH8Gipec8Eeeu0xXdbba9frFj0=OqFfea0dXdd9vqai=hGuQ8kuc9pgc9s8qqaq=dirpe0xb9q8qiLsFr0=vr0=vr0dc8meaabaqaciaacaGaaeqabaqabeGadaaakeaacqWGtbWucqWGdbWqdaWgaaWcbaGaemiDaqNaemOCaiNaemyAaKMaemiCaaNaemiBaWMaemyzauMaemiDaqhabeaakiabcIcaOiabdsfaunaaCaaaleqabaGaemyCaehaaOGaeiilaWIaemivaq1aaWbaaSqabeaacqWG0baDaaGccqGGPaqkcqGH9aqpcqWGtbWucqWGdbWqdaWgaaWcbaGaemiCaaNaemyyaeMaemyAaKMaemOCaihabeaakiabcIcaOiabdofatnaaDaaaleaacqWGPbqAcqWGQbGAaeaacqWGXbqCaaGccqGGSaalcqWGtbWudaqhaaWcbaGaemyAaKMaemOAaOgabaGaemiDaqhaaOGaeiykaKIaey4kaSIaem4uamLaem4qam0aaSbaaSqaaiabdchaWjabdggaHjabdMgaPjabdkhaYbqabaGccqGGOaakcqWGtbWudaqhaaWcbaGaemOAaOMaem4AaSgabaGaemyCaehaaOGaeiilaWIaem4uam1aa0baaSqaaiabdQgaQjabdUgaRbqaaiabdsha0baakiabcMcaPiabgUcaRiabdofatjabdoeadnaaBaaaleaacqWGWbaCcqWGHbqycqWGPbqAcqWGYbGCaeqaaOGaeiikaGIaem4uam1aa0baaSqaaiabdMgaPjabdUgaRbqaaiabdghaXbaakiabcYcaSiabdofatnaaDaaaleaacqWGPbqAcqWGRbWAaeaacqWG0baDaaGccqGGPaqkaaa@84C9@

where Sijq
 MathType@MTEF@5@5@+=feaafiart1ev1aaatCvAUfKttLearuWrP9MDH5MBPbIqV92AaeXatLxBI9gBaebbnrfifHhDYfgasaacH8akY=wiFfYdH8Gipec8Eeeu0xXdbba9frFj0=OqFfea0dXdd9vqai=hGuQ8kuc9pgc9s8qqaq=dirpe0xb9q8qiLsFr0=vr0=vr0dc8meaabaqaciaacaGaaeqabaqabeGadaaakeaacqWGtbWudaqhaaWcbaGaemyAaKMaemOAaOgabaGaemyCaehaaaaa@322B@ and Sijt
 MathType@MTEF@5@5@+=feaafiart1ev1aaatCvAUfKttLearuWrP9MDH5MBPbIqV92AaeXatLxBI9gBaebbnrfifHhDYfgasaacH8akY=wiFfYdH8Gipec8Eeeu0xXdbba9frFj0=OqFfea0dXdd9vqai=hGuQ8kuc9pgc9s8qqaq=dirpe0xb9q8qiLsFr0=vr0=vr0dc8meaabaqaciaacaGaaeqabaqabeGadaaakeaacqWGtbWudaqhaaWcbaGaemyAaKMaemOAaOgabaGaemiDaqhaaaaa@3231@ denote the equivalent SSE pairs of *S*_*i *_and *S*_*j*_

The score, *SC*_*pair*_(Sijq
 MathType@MTEF@5@5@+=feaafiart1ev1aaatCvAUfKttLearuWrP9MDH5MBPbIqV92AaeXatLxBI9gBaebbnrfifHhDYfgasaacH8akY=wiFfYdH8Gipec8Eeeu0xXdbba9frFj0=OqFfea0dXdd9vqai=hGuQ8kuc9pgc9s8qqaq=dirpe0xb9q8qiLsFr0=vr0=vr0dc8meaabaqaciaacaGaaeqabaqabeGadaaakeaacqWGtbWudaqhaaWcbaGaemyAaKMaemOAaOgabaGaemyCaehaaaaa@322B@, Sijt
 MathType@MTEF@5@5@+=feaafiart1ev1aaatCvAUfKttLearuWrP9MDH5MBPbIqV92AaeXatLxBI9gBaebbnrfifHhDYfgasaacH8akY=wiFfYdH8Gipec8Eeeu0xXdbba9frFj0=OqFfea0dXdd9vqai=hGuQ8kuc9pgc9s8qqaq=dirpe0xb9q8qiLsFr0=vr0=vr0dc8meaabaqaciaacaGaaeqabaqabeGadaaakeaacqWGtbWudaqhaaWcbaGaemyAaKMaemOAaOgabaGaemiDaqhaaaaa@3231@), is the SSE pair similarity score, and is computed as:

SCpair(Sijq,Sijt)={θE−|Dijq−Dijt|Dij∗}×w(Dij∗)×(li×lj)p
 MathType@MTEF@5@5@+=feaafiart1ev1aaatCvAUfKttLearuWrP9MDH5MBPbIqV92AaeXatLxBI9gBaebbnrfifHhDYfgasaacH8akY=wiFfYdH8Gipec8Eeeu0xXdbba9frFj0=OqFfea0dXdd9vqai=hGuQ8kuc9pgc9s8qqaq=dirpe0xb9q8qiLsFr0=vr0=vr0dc8meaabaqaciaacaGaaeqabaqabeGadaaakeaacqWGtbWucqWGdbWqdaWgaaWcbaGaemiCaaNaemyyaeMaemyAaKMaemOCaihabeaakiabcIcaOiabdofatnaaDaaaleaacqWGPbqAcqWGQbGAaeaacqWGXbqCaaGccqGGSaalcqWGtbWudaqhaaWcbaGaemyAaKMaemOAaOgabaGaemiDaqhaaOGaeiykaKIaeyypa0ZaaiWabeaaiiGacqWF4oqCdaahaaWcbeqaaiabdweafbaakiabgkHiTmaalaaabaGaeiiFaWNaemiraq0aa0baaSqaaiabdMgaPjabdQgaQbqaaiabdghaXbaakiabgkHiTiabdseaenaaDaaaleaacqWGPbqAcqWGQbGAaeaacqWG0baDaaGccqGG8baFaeaacqWGebardaqhaaWcbaGaemyAaKMaemOAaOgabaGaey4fIOcaaaaaaOGaay5Eaiaaw2haaiabgEna0kabdEha3jabcIcaOiabdseaenaaDaaaleaacqWGPbqAcqWGQbGAaeaacqGHxiIkaaGccqGGPaqkcqGHxdaTcqGGOaakcqWGSbaBdaWgaaWcbaGaemyAaKgabeaakiabgEna0kabdYgaSnaaBaaaleaacqWGQbGAaeqaaOGaeiykaKYaaWbaaSqabeaacqWGWbaCaaaaaa@74C5@

where Dij∗
 MathType@MTEF@5@5@+=feaafiart1ev1aaatCvAUfKttLearuWrP9MDH5MBPbIqV92AaeXatLxBI9gBaebbnrfifHhDYfgasaacH8akY=wiFfYdH8Gipec8Eeeu0xXdbba9frFj0=OqFfea0dXdd9vqai=hGuQ8kuc9pgc9s8qqaq=dirpe0xb9q8qiLsFr0=vr0=vr0dc8meaabaqaciaacaGaaeqabaqabeGadaaakeaacqWGebardaqhaaWcbaGaemyAaKMaemOAaOgabaGaey4fIOcaaaaa@3191@ is the average of Dijq
 MathType@MTEF@5@5@+=feaafiart1ev1aaatCvAUfKttLearuWrP9MDH5MBPbIqV92AaeXatLxBI9gBaebbnrfifHhDYfgasaacH8akY=wiFfYdH8Gipec8Eeeu0xXdbba9frFj0=OqFfea0dXdd9vqai=hGuQ8kuc9pgc9s8qqaq=dirpe0xb9q8qiLsFr0=vr0=vr0dc8meaabaqaciaacaGaaeqabaqabeGadaaakeaacqWGebardaqhaaWcbaGaemyAaKMaemOAaOgabaGaemyCaehaaaaa@320D@ and Dijt
 MathType@MTEF@5@5@+=feaafiart1ev1aaatCvAUfKttLearuWrP9MDH5MBPbIqV92AaeXatLxBI9gBaebbnrfifHhDYfgasaacH8akY=wiFfYdH8Gipec8Eeeu0xXdbba9frFj0=OqFfea0dXdd9vqai=hGuQ8kuc9pgc9s8qqaq=dirpe0xb9q8qiLsFr0=vr0=vr0dc8meaabaqaciaacaGaaeqabaqabeGadaaakeaacqWGebardaqhaaWcbaGaemyAaKMaemOAaOgabaGaemiDaqhaaaaa@3213@*w*(*r*) = exp(-(*r*/20)^2^), *l*_*i *_= min(Liq
 MathType@MTEF@5@5@+=feaafiart1ev1aaatCvAUfKttLearuWrP9MDH5MBPbIqV92AaeXatLxBI9gBaebbnrfifHhDYfgasaacH8akY=wiFfYdH8Gipec8Eeeu0xXdbba9frFj0=OqFfea0dXdd9vqai=hGuQ8kuc9pgc9s8qqaq=dirpe0xb9q8qiLsFr0=vr0=vr0dc8meaabaqaciaacaGaaeqabaqabeGadaaakeaacqWGmbatdaqhaaWcbaGaemyAaKgabaGaemyCaehaaaaa@30C0@, Lit
 MathType@MTEF@5@5@+=feaafiart1ev1aaatCvAUfKttLearuWrP9MDH5MBPbIqV92AaeXatLxBI9gBaebbnrfifHhDYfgasaacH8akY=wiFfYdH8Gipec8Eeeu0xXdbba9frFj0=OqFfea0dXdd9vqai=hGuQ8kuc9pgc9s8qqaq=dirpe0xb9q8qiLsFr0=vr0=vr0dc8meaabaqaciaacaGaaeqabaqabeGadaaakeaacqWGmbatdaqhaaWcbaGaemyAaKgabaGaemiDaqhaaaaa@30C6@), *l*_*j *_= min(Ljq
 MathType@MTEF@5@5@+=feaafiart1ev1aaatCvAUfKttLearuWrP9MDH5MBPbIqV92AaeXatLxBI9gBaebbnrfifHhDYfgasaacH8akY=wiFfYdH8Gipec8Eeeu0xXdbba9frFj0=OqFfea0dXdd9vqai=hGuQ8kuc9pgc9s8qqaq=dirpe0xb9q8qiLsFr0=vr0=vr0dc8meaabaqaciaacaGaaeqabaqabeGadaaakeaacqWGmbatdaqhaaWcbaGaemOAaOgabaGaemyCaehaaaaa@30C2@, Ljt
 MathType@MTEF@5@5@+=feaafiart1ev1aaatCvAUfKttLearuWrP9MDH5MBPbIqV92AaeXatLxBI9gBaebbnrfifHhDYfgasaacH8akY=wiFfYdH8Gipec8Eeeu0xXdbba9frFj0=OqFfea0dXdd9vqai=hGuQ8kuc9pgc9s8qqaq=dirpe0xb9q8qiLsFr0=vr0=vr0dc8meaabaqaciaacaGaaeqabaqabeGadaaakeaacqWGmbatdaqhaaWcbaGaemOAaOgabaGaemiDaqhaaaaa@30C8@), *p *= 0.6, and *θ*^*E *^= 0.2.

In the above equation, the first term measures the distance deviation between two SSE pairs. The second term de-emphasizes the significance of matches between two distant SSE pairs, since "distant SSE pairs are abundant and less discriminate" [24]. The last term scales the score by the maximum aligned portion between the SSE pairs and a parameter *p*. The parameter *p *is set to 0.6, which was empirically determined by randomly choosing 400 domains from ASTRAL and computing our *SC*_*pair *_score and DALI score for each SSE pair. The value of *p *= 0.6 produced the maximum correlation between the two scores (correlation coefficient of 0.6).

We note that our scoring equation has a strong similarity to the DALI scoring model. In the DALI model, a similarity score is calculated using all pairwise residue distances, whereas in our model the basic unit of comparison is an SSE rather than individual residues. The scoring using the SSE uses only the information in the index, and is computationally much faster than the scoring function used in DALI (which costs *O*(*N*^2^) where *N *is the number of residues).

##### SSE triplet index search

When matching a query triplet, rather than scanning all the SSE triplets in the database (which can be slow), we use an index search to find all database triplets that are similar to the query triplet. For each database triplet, we then compute the similarity score with the query triplet using the *SC*_*triplet *_equation described below.

The index probe retrieves all matching entries using the following criteria: Given a query triplet *T*^*q *^and a target triplet T^*t*^, which are defined as below,

Tq=(TCq,Xiq,Yiq,Xjq,Yjq,Xkq,Ykq,Djkq,Dikq,Dijq)Tt=(TCt,Xit,Yit,Xjt,Yjt,Xkt,Ykt,Djkt,Dikt,Dijt)
 MathType@MTEF@5@5@+=feaafiart1ev1aaatCvAUfKttLearuWrP9MDH5MBPbIqV92AaeXatLxBI9gBaebbnrfifHhDYfgasaacH8akY=wiFfYdH8Gipec8Eeeu0xXdbba9frFj0=OqFfea0dXdd9vqai=hGuQ8kuc9pgc9s8qqaq=dirpe0xb9q8qiLsFr0=vr0=vr0dc8meaabaqaciaacaGaaeqabaqabeGadaaakqaaeeqaaiabdsfaunaaCaaaleqabaGaemyCaehaaOGaeyypa0JaeiikaGIaemivaqLaem4qam0aaWbaaSqabeaacqWGXbqCaaGccqGGSaalcqWGybawdaqhaaWcbaGaemyAaKgabaGaemyCaehaaOGaeiilaWIaemywaK1aa0baaSqaaiabdMgaPbqaaiabdghaXbaakiabcYcaSiabdIfaynaaDaaaleaacqWGQbGAaeaacqWGXbqCaaGccqGGSaalcqWGzbqwdaqhaaWcbaGaemOAaOgabaGaemyCaehaaOGaeiilaWIaemiwaG1aa0baaSqaaiabdUgaRbqaaiabdghaXbaakiabcYcaSiabdMfaznaaDaaaleaacqWGRbWAaeaacqWGXbqCaaGccqGGSaalcqWGebardaqhaaWcbaGaemOAaOMaem4AaSgabaGaemyCaehaaOGaeiilaWIaemiraq0aa0baaSqaaiabdMgaPjabdUgaRbqaaiabdghaXbaakiabcYcaSiabdseaenaaDaaaleaacqWGPbqAcqWGQbGAaeaacqWGXbqCaaGccqGGPaqkaeaacqWGubavdaahaaWcbeqaaiabdsha0baakiabg2da9iabcIcaOiabdsfaujabdoeadnaaCaaaleqabaGaemiDaqhaaOGaeiilaWIaemiwaG1aa0baaSqaaiabdMgaPbqaaiabdsha0baakiabcYcaSiabdMfaznaaDaaaleaacqWGPbqAaeaacqWG0baDaaGccqGGSaalcqWGybawdaqhaaWcbaGaemOAaOgabaGaemiDaqhaaOGaeiilaWIaemywaK1aa0baaSqaaiabdQgaQbqaaiabdsha0baakiabcYcaSiabdIfaynaaDaaaleaacqWGRbWAaeaacqWG0baDaaGccqGGSaalcqWGzbqwdaqhaaWcbaGaem4AaSgabaGaemiDaqhaaOGaeiilaWIaemiraq0aa0baaSqaaiabdQgaQjabdUgaRbqaaiabdsha0baakiabcYcaSiabdseaenaaDaaaleaacqWGPbqAcqWGRbWAaeaacqWG0baDaaGccqGGSaalcqWGebardaqhaaWcbaGaemyAaKMaemOAaOgabaGaemiDaqhaaOGaeiykaKcaaaa@A2CC@

T^*t *^is a match for *T*^*q *^when the following conditions are met.

1.TCq=TCt,2.∀r=i,j,k((Xrq−Xrt)2+(Yrq−Yrt)2≤sin(θ)×Xrq+Yrq),  and3.|Djkq−Djkt| ≤dε1Λ|Dikq−Dikt| ≤dε2Λ|Dijq−Dijt| ≤dε3
 MathType@MTEF@5@5@+=feaafiart1ev1aaatCvAUfKttLearuWrP9MDH5MBPbIqV92AaeXatLxBI9gBaebbnrfifHhDYfgasaacH8akY=wiFfYdH8Gipec8Eeeu0xXdbba9frFj0=OqFfea0dXdd9vqai=hGuQ8kuc9pgc9s8qqaq=dirpe0xb9q8qiLsFr0=vr0=vr0dc8meaabaqaciaacaGaaeqabaqabeGadaaakqaabeqaaiabigdaXiabc6caUiabdsfaujabdoeadnaaCaaaleqabaGaemyCaehaaOGaeyypa0JaemivaqLaem4qam0aaWbaaSqabeaacqWG0baDaaGccqGGSaalaeaacqaIYaGmcqGGUaGlcqGHaiIicqWGYbGCcqGH9aqpcqWGPbqAcqGGSaalcqWGQbGAcqGGSaalcqWGRbWAcqGGOaakdaGcaaqaaiabcIcaOiabdIfaynaaDaaaleaacqWGYbGCaeaacqWGXbqCaaGccqGHsislcqWGybawdaqhaaWcbaGaemOCaihabaGaemiDaqhaaOGaeiykaKYaaWbaaSqabeaacqaIYaGmaaGccqGHRaWkcqGGOaakcqWGzbqwdaqhaaWcbaGaemOCaihabaGaemyCaehaaOGaeyOeI0IaemywaK1aa0baaSqaaiabdkhaYbqaaiabdsha0baakiabcMcaPmaaCaaaleqabaGaeGOmaidaaaqabaGccqGHKjYOieGacqWFZbWCcqWFPbqAcqWFUbGBcqGGOaakiiGacqGF4oqCcqGGPaqkcqGHxdaTdaGcaaqaaiabdIfaynaaDaaaleaacqWGYbGCaeaacqWGXbqCaaGccqGHRaWkcqWGzbqwdaqhaaWcbaGaemOCaihabaGaemyCaehaaaqabaGccqGGPaqkcqGGSaalcaaMc8UaaGPaVlabbggaHjabb6gaUjabbsgaKbqaaiabiodaZiabc6caUiabcYha8jabdseaenaaDaaaleaacqWGQbGAcqWGRbWAaeaacqWGXbqCaaGccqGHsislcqWGebardaqhaaWcbaGaemOAaOMaem4AaSgabaGaemiDaqhaaOGaeiiFaWNaaGPaVlabgsMiJkadmc4GKbazdGaJaUbaaSqaiWiGcWaJa6xTduMamWiGigdaXaqajWiGaOGamWiGfU5amjabcYha8jabdseaenaaDaaaleaacqWGPbqAcqWGRbWAaeaacqWGXbqCaaGccqGHsislcqWGebardaqhaaWcbaGaemyAaKMaem4AaSgabaGaemiDaqhaaOGaeiiFaWNaaGPaVlabgsMiJkabdsgaKnaaBaaaleaacqGF1oqzcqaIYaGmaeqaaOGaeu4MdWKaeiiFaWNaemiraq0aa0baaSqaaiabdMgaPjabdQgaQbqaaiabdghaXbaakiabgkHiTiabdseaenaaDaaaleaacqWGPbqAcqWGQbGAaeaacqWG0baDaaGccqGG8baFcaaMc8UaeyizImQaemizaq2aaSbaaSqaaiab+v7aLjabiodaZaqabaaaaaa@CA22@

The first condition checks to ensure that the two SSE triplets have the same SSE types and the same order for the SSEs. The second condition checks to see if the three SSEs in *T*^*t *^are within in a small distance (*sin*(*θ*) × Xrq+Yrq
 MathType@MTEF@5@5@+=feaafiart1ev1aaatCvAUfKttLearuWrP9MDH5MBPbIqV92AaeXatLxBI9gBaebbnrfifHhDYfgasaacH8akY=wiFfYdH8Gipec8Eeeu0xXdbba9frFj0=OqFfea0dXdd9vqai=hGuQ8kuc9pgc9s8qqaq=dirpe0xb9q8qiLsFr0=vr0=vr0dc8meaabaqaciaacaGaaeqabaqabeGadaaakeaadaGcaaqaaiabdIfaynaaDaaaleaacqWGYbGCaeaacqWGXbqCaaGccqGHRaWkcqWGzbqwdaqhaaWcbaGaemOCaihabaGaemyCaehaaaqabaaaaa@3626@) of the corresponding SSEs in *T*^*q*^. In our implementation, *θ *is set to 30°. The final condition checks if the distance between each matching SSE pair is within a small threshold, *d*_*ε*_. As in the DALI scoring model [24], the exact value for this threshold depends on the types of the SSEs being compared. The distance cutoff is set to 3 Å for a *β*-strand pair, 4 Å for an *α*-helix and *β*-strand pair, and 5 Å for an *α *helix pair. We note that these cutoff values are higher than the ones used in the DALI model as we are matching SSEs in a triplet, rather than just individual pair without considering a triplet configuration (as is done in DALI). The original DALI cutoffs would be too strict for matching SSE triplets.

#### Protein structure matching

The previous step produces matching target triplets in the database for every triplet in the query, and the associated matching score (*SC*_*triplet*_). Next, we need to assemble these triplet hits into matches for the entire protein domain. For this step, we construct a weighted bipartite graph for *every *target protein domain that has some triplet matches. In each graph, nodes on one side of the bipartite graph represent triplets in the query and nodes on the other side represent triplets in the database entry. An edge between two nodes indicates that the two triplets were matched by the previous step, and the weight of the edge represents the *SC*_*triplet *_score. A maximum weighted bipartite graph matching algorithm is run on this graph to produce an injective (one-to-one) mapping from the query SSE triplets to the triplets in the target. Then, using this mapping, an overall structure similarity score is computed as:

SCraw(q,t)=∑i=1MSCtriplet(Tiq,Tit)
 MathType@MTEF@5@5@+=feaafiart1ev1aaatCvAUfKttLearuWrP9MDH5MBPbIqV92AaeXatLxBI9gBaebbnrfifHhDYfgasaacH8akY=wiFfYdH8Gipec8Eeeu0xXdbba9frFj0=OqFfea0dXdd9vqai=hGuQ8kuc9pgc9s8qqaq=dirpe0xb9q8qiLsFr0=vr0=vr0dc8meaabaqaciaacaGaaeqabaqabeGadaaakeaacqWGtbWucqWGdbWqdaWgaaWcbaGaemOCaiNaemyyaeMaem4DaChabeaakiabcIcaOiabdghaXjabcYcaSiabdsha0jabcMcaPiabg2da9maaqadabaGaem4uamLaem4qam0aaSbaaSqaaiabdsha0jabdkhaYjabdMgaPjabdchaWjabdYgaSjabdwgaLjabdsha0bqabaGccqGGOaakcqWGubavdaqhaaWcbaGaemyAaKgabaGaemyCaehaaOGaeiilaWIaemivaq1aa0baaSqaaiabdMgaPbqaaiabdsha0baakiabcMcaPaWcbaGaemyAaKMaeyypa0JaeGymaedabaGaemyta0eaniabggHiLdaaaa@5768@

where Tiq
 MathType@MTEF@5@5@+=feaafiart1ev1aaatCvAUfKttLearuWrP9MDH5MBPbIqV92AaeXatLxBI9gBaebbnrfifHhDYfgasaacH8akY=wiFfYdH8Gipec8Eeeu0xXdbba9frFj0=OqFfea0dXdd9vqai=hGuQ8kuc9pgc9s8qqaq=dirpe0xb9q8qiLsFr0=vr0=vr0dc8meaabaqaciaacaGaaeqabaqabeGadaaakeaacqWGubavdaqhaaWcbaGaemyAaKgabaGaemyCaehaaaaa@30D0@ and Tit
 MathType@MTEF@5@5@+=feaafiart1ev1aaatCvAUfKttLearuWrP9MDH5MBPbIqV92AaeXatLxBI9gBaebbnrfifHhDYfgasaacH8akY=wiFfYdH8Gipec8Eeeu0xXdbba9frFj0=OqFfea0dXdd9vqai=hGuQ8kuc9pgc9s8qqaq=dirpe0xb9q8qiLsFr0=vr0=vr0dc8meaabaqaciaacaGaaeqabaqabeGadaaakeaacqWGubavdaqhaaWcbaGaemyAaKgabaGaemiDaqhaaaaa@30D6@ are equivalent SSE triplets in the one-to-one mapping, *M *is the total number of equivalent SSE triplet pairs, and *SC*_*triplet*_(Tiq
 MathType@MTEF@5@5@+=feaafiart1ev1aaatCvAUfKttLearuWrP9MDH5MBPbIqV92AaeXatLxBI9gBaebbnrfifHhDYfgasaacH8akY=wiFfYdH8Gipec8Eeeu0xXdbba9frFj0=OqFfea0dXdd9vqai=hGuQ8kuc9pgc9s8qqaq=dirpe0xb9q8qiLsFr0=vr0=vr0dc8meaabaqaciaacaGaaeqabaqabeGadaaakeaacqWGubavdaqhaaWcbaGaemyAaKgabaGaemyCaehaaaaa@30D0@, Tit
 MathType@MTEF@5@5@+=feaafiart1ev1aaatCvAUfKttLearuWrP9MDH5MBPbIqV92AaeXatLxBI9gBaebbnrfifHhDYfgasaacH8akY=wiFfYdH8Gipec8Eeeu0xXdbba9frFj0=OqFfea0dXdd9vqai=hGuQ8kuc9pgc9s8qqaq=dirpe0xb9q8qiLsFr0=vr0=vr0dc8meaabaqaciaacaGaaeqabaqabeGadaaakeaacqWGubavdaqhaaWcbaGaemyAaKgabaGaemiDaqhaaaaa@30D6@) is the triplet similarity score between Tiq
 MathType@MTEF@5@5@+=feaafiart1ev1aaatCvAUfKttLearuWrP9MDH5MBPbIqV92AaeXatLxBI9gBaebbnrfifHhDYfgasaacH8akY=wiFfYdH8Gipec8Eeeu0xXdbba9frFj0=OqFfea0dXdd9vqai=hGuQ8kuc9pgc9s8qqaq=dirpe0xb9q8qiLsFr0=vr0=vr0dc8meaabaqaciaacaGaaeqabaqabeGadaaakeaacqWGubavdaqhaaWcbaGaemyAaKgabaGaemyCaehaaaaa@30D0@ and Tit
 MathType@MTEF@5@5@+=feaafiart1ev1aaatCvAUfKttLearuWrP9MDH5MBPbIqV92AaeXatLxBI9gBaebbnrfifHhDYfgasaacH8akY=wiFfYdH8Gipec8Eeeu0xXdbba9frFj0=OqFfea0dXdd9vqai=hGuQ8kuc9pgc9s8qqaq=dirpe0xb9q8qiLsFr0=vr0=vr0dc8meaabaqaciaacaGaaeqabaqabeGadaaakeaacqWGubavdaqhaaWcbaGaemyAaKgabaGaemiDaqhaaaaa@30D6@. This raw similarity score depends on the sizes of the query and target protein domains, and is normalized as follows:

SCnorm(q,t)=(SCraw(q,t)×R*)/SCraw(q,q), whereR*=RTotal(q,t)+RSSE(q,t)2,RTotal(q,t)=1+max⁡(−1,log⁡10Nqmax⁡(Nq,Nt)), andRSSE(q,t)=1+max⁡(−1,log⁡10SNtmax⁡(SNq,SNt))
 MathType@MTEF@5@5@+=feaafiart1ev1aaatCvAUfKttLearuWrP9MDH5MBPbIqV92AaeXatLxBI9gBaebbnrfifHhDYfgasaacH8akY=wiFfYdH8Gipec8Eeeu0xXdbba9frFj0=OqFfea0dXdd9vqai=hGuQ8kuc9pgc9s8qqaq=dirpe0xb9q8qiLsFr0=vr0=vr0dc8meaabaqaciaacaGaaeqabaqabeGadaaakqaabeqaaiabdofatjabdoeadnaaBaaaleaacqWGUbGBcqWGVbWBcqWGYbGCcqWGTbqBaeqaaOGaeiikaGIaemyCaeNaeiilaWIaemiDaqNaeiykaKIaeyypa0JaeiikaGIaem4uamLaem4qam0aaSbaaSqaaiabdkhaYjabdggaHjabdEha3bqabaGccqGGOaakcqWGXbqCcqGGSaalcqWG0baDcqGGPaqkcqGHxdaTcqWGsbGucqGGQaGkcqGGPaqkcqGGVaWlcqWGtbWucqWGdbWqdaWgaaWcbaGaemOCaiNaemyyaeMaem4DaChabeaakiabcIcaOiabdghaXjabcYcaSiabdghaXjabcMcaPiabcYcaSiaaykW7cqqG3bWDcqqGObaAcqqGLbqzcqqGYbGCcqqGLbqzaeaacqWGsbGucqGGQaGkcqGH9aqpdaWcaaqaaiabdkfasnaaBaaaleaacqWGubavcqWGVbWBcqWG0baDcqWGHbqycqWGSbaBaeqaaOGaeiikaGIaemyCaeNaeiilaWIaemiDaqNaeiykaKIaey4kaSIaemOuai1aaSbaaSqaaiabdofatjabdofatjabdweafbqabaGccqGGOaakcqWGXbqCcqGGSaalcqWG0baDcqGGPaqkaeaacqaIYaGmaaGaeiilaWcabaGaemOuai1aaSbaaSqaaiabdsfaujabd+gaVjabdsha0jabdggaHjabdYgaSbqabaGccqGGOaakcqWGXbqCcqGGSaalcqWG0baDcqGGPaqkcqGH9aqpcqaIXaqmcqGHRaWkcyGGTbqBcqGGHbqycqGG4baEdaqadaqaaiabgkHiTiabigdaXiabcYcaSiGbcYgaSjabc+gaVjabcEgaNnaaBaaaleaacqaIXaqmcqaIWaamaeqaaOWaaSaaaeaacqWGobGtdaWgaaWcbaGaemyCaehabeaaaOqaaiGbc2gaTjabcggaHjabcIha4jabcIcaOiabd6eaonaaBaaaleaacqWGXbqCaeqaaOGaeiilaWIaemOta40aaSbaaSqaaiabdsha0bqabaGccqGGPaqkaaaacaGLOaGaayzkaaGaeiilaWIaaGPaVlabbggaHjabb6gaUjabbsgaKbqaaiabdkfasnaaBaaaleaacqWGtbWucqWGtbWucqWGfbqraeqaaOGaeiikaGIaemyCaeNaeiilaWIaemiDaqNaeiykaKIaeyypa0JaeGymaeJaey4kaSIagiyBa0MaeiyyaeMaeiiEaG3aaeWaaeaacqGHsislcqaIXaqmcqGGSaalcyGGSbaBcqGGVbWBcqGGNbWzdaWgaaWcbaGaeGymaeJaeGimaadabeaakmaalaaabaGaem4uamLaemOta40aaSbaaSqaaiabdsha0bqabaaakeaacyGGTbqBcqGGHbqycqGG4baEcqGGOaakcqWGtbWucqWGobGtdaWgaaWcbaGaemyCaehabeaakiabcYcaSiabdofatjabd6eaonaaBaaaleaacqWG0baDaeqaaOGaeiykaKcaaaGaayjkaiaawMcaaaaaaa@E3D9@

In the equations above, *N*_*q *_and *N*_*t *_are the total number of residues in the query and the target respectively. *SN*_*q *_and *SN*_*t *_are the number of residues contributing to the formation of *α*-helices and *β*-strands in the query and the target respectively. The ratios, *R*_*Total *_and *R*_*SSE*_, scale down the raw score inversely proportional to the size difference between the two proteins, and produce a score that is less sensitive to the size differences between the query and the target. The division by the self-similarity score, *SC*_*raw*_(*q*, *q*) produces a normalized similarity score between 0 and 1, which represents how similar the query protein is to a target, compared to itself. The normalized score is reported as the final structure similarity score.

### Structure classification

Existing automatic classification methods [5,7-9] employ a nearest neighbor classification strategy. Given a query protein domain, they find the structurally closest neighbor that has a known classification label. Then the query is assigned the same label as its nearest neighbor. Although the nearest classification strategy is effective in many cases, it has a significant limitation as proteins with novel folds are guaranteed to be misclassified.

To resolve this problem, the SGM method [7], which employs a modified nearest-neighbor approach, reports a label of *unknown and/or possibly new *when it cannot classify proteins with high confidence. To detect the boundary between classification and non-classification, it uses an inter to intra cluster distance ratio, based on the observation that "chains that are equidistant to several clusters are hard to classify and chains that are far away from any known clusters are probably new folds" [7]. The distance ratio can be effectively used to detect whether a protein is *relatively *closer to a specific cluster than to the remaining clusters. However, it cannot be used to detect whether a protein is *absolutely *close to a specific cluster.

Our classification method is also based on the nearest neighbor classification, and adopts the same observation as is used in SGM to detect unknown and/or possibly new folds. However, our method improves classification accuracy by using additional measures and a more sophisticated class boundary detection method, namely an SVM [21]. Furthermore, instead of reporting proteins labels as "unknown and/or possibly new", our method also identifies clusters among unclassified proteins to further automate the classification process.

#### Classification using an SVM

Our method for assigning a class label uses three pieces of information, namely: an absolute similarity ratio (*F*1), a relative similarity ratio (*F*2), and the nearest cluster classification label (*C*1). This information is collected using the following procedure:

First, given a protein domain *q*, the structure comparison method, described in the structure comparison section, is used to find the top *k *structure neighbors in the database. From this top *k *list, we remove any hits to the query itself.

Then, we pick the top structure, *n*_1 _as the nearest neighbor. Let *C*1 denote *n*_1_'s classification label. We then go down the list and find the next structure that has a different label from *C*1. Let us call this entry *n*_2_, and let *C*2 denote the label for *n*_2 _. Next, we compute the scores, *SC*_*norm*_(*q*, *n*_1_) and *SC*_*norm*_(*q*, *n*_2_).

Then we use these scores to compute *F*1 and *F*2 as: *F*1 = *SC*_*norm*_(*q*, *n*_1_) and *F*2 = *SC*_*norm*_(*q*, *n*_1_)/*SC*_*norm*_(*q*, *n*_2_). Finally, we return the values *F*1, *F*2 and *C*1.

Intuitively, high *F*1 and *F*2 values indicate that the query is structurally similar to its nearest neighbor, and is also relatively closer to its nearest neighbor than to any other domain, which in turn suggests a high confidence in the assignment of the classification label. On the other hand, low *F*1 and *F*2 values imply that the domain is not particularly similar to any existing domains, which suggests that the domain is potentially a new fold.

**Figure 2:**
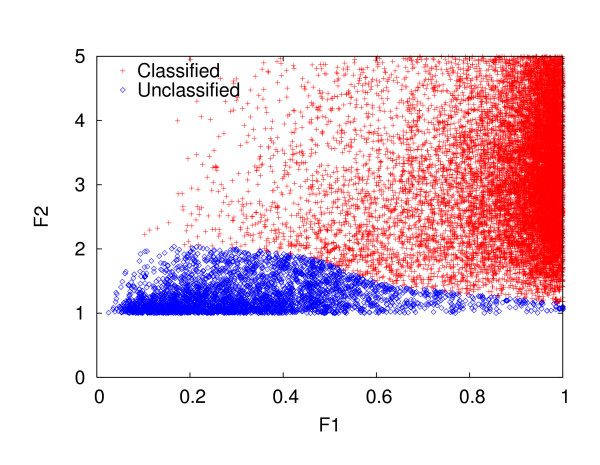
**Visualization of the classification decision boundary**. This figure shows the classification boundary created for entries in SCOP 1.67 using SCOP 1.65 as the database. The SVM is used to detect the boundary between "Classified" and "Unclassified" entries. This trained SVM will then be used to predict class labels for SCOP 1.69.

To automate the classification process, we need a classification decision model which defines clear boundaries between classification and non-classification. Using SCOP as the gold standard, we generated a classification decision model that reflects the rules used for creating new folds in SCOP. In order to create such classification decision model, we used a support vector machine (SVM) to capture nonlinear classification decision boundaries in SCOP. As a training set for the decision model, we picked SCOP version 1.65 and version 1.67 and trained the model as follows: Using domains in SCOP 1.67 as the queries, and domains in SCOP 1.65 as the database, we perform structure comparison using the method described in the structure comparison section. For each query, we calculate the *F*1 and *F*2 scores. If the SCOP label of a query protein domain is the same as its nearest neighbor's SCOP label, the query with its *F*1 and *F*2 is used as a positive example, otherwise, it is used as a negative example in the training set for the SVM. The resulting training dataset is shown in Figure 2.

The classification label assignment step simply uses the trained SVM to determine if a query should be labeled as *unclassified*. For queries that the SVM determines can be classified, the label of the nearest-neighbor in the database is used as the predicted class label.

### Identification and clustering of novel structures

Our classification method takes the approach of assigning an "unclassified" label to protein domains that have novel folds or have subjective and fuzzy classification boundaries. Assigning an actual class label to such domains often requires additional biological information and manual interpretation [1,29]. Since such manual intervention is likely to continue to be unavoidable even in the foreseeable future, it is useful if additional information is provided to make a more informed (and potentially faster) manual assignment. In this section, we outline our method for aiding this manual assignment process by employing a clustering method for grouping the protein domains that are labeled as "unclassified" with our classification method. The basic intuition behind using clustering is that protein domains that are in the same cluster are likely to have stronger similarities to each other, sharing similar protein structures, compared to domains in different clusters. In addition, it is often likely that well-segregated clusters correspond to novel folds. To detect these novel folds, we first perform an all-to-all comparison using all the protein domains that are labeled as unclassified by the previous structure classification step. Then, we construct a graph that has a node for every unclassified domain. In this graph two nodes are connected by an edge if the similarity score between the protein domains corresponding to the nodes is above a certain threshold. Each edge has a weight, which is equal to the similarity score. Once this graph is constructed, the MCL [30] algorithm is run on the graph to detect clusters. (MCL is a clustering algorithm that is specifically designed to work with graphs.) The computed clusters are then reported as groups that potentially correspond to novel folds. In addition, for each computed cluster we also produce a representative structure, which is simply the graph center for that cluster (if there are more than one centers, we randomly select one of the centers as the representative structure).

## Availability

A web site offering access for protein classification is freely available at .

## Authors' contributions

YJ carried out the design, implementation, and evaluation of proCC, and drafted the manuscript. JP conceived of the study, participated in its design and evaluation, and helped to draft the manuscript. All authors read and approved the final manuscript.

## Supplementary Material

Additional File 1 **roccurves**. In this supplementary material, we present results evaluating the structure comparison methods using ROC curves. Click here for file
