# Supplementary material for: A framework for protein structure classification and identification of novel protein structures
Source: BMC Bioinformatics. 2006 Oct 16;7:456. doi: 10.1186/1471-2105-7-456 (PMC1622760; doi:10.1186/1471-2105-7-456)
Supplement: Additional File 1 — roccurves. In this supplementary material, we present results evaluating the structure comparison methods using ROC curves. [file 1471-2105-7-456-S1.pdf]

Evaluation of structure comparison methods

In this supplementary material, we present results evaluating structure comparison methods using ROC curves. We compare proCC with SGM to evaluate their effectiveness in finding homologous domains. We note that we compare proCC only with SGM for this ROC benchmark. Comparison with SCOPmap is missing in this benchmarking experiment, since SCOPmap only returns one classification label for a given query (and does not produce a list of nearest structures to the query which is required to produce an ROC curve).

We produce the ROC curves as follows (mirroring the method used by Wallqvist et al.[1]): New domain structures introduced in SCOP 1.69, excluding new domains which do not have any structural neighbors in SCOP 1.67, are used as queries. These query domains are then searched against a database containing domains in SCOP 1.67. The search produces pairs of query and target domains. The query and target pairs are sorted with the best matches at the top of the list. (Pairs are sorted from high to low scores in our method and from low to high distances in the SGM method.) Then, for each pair, the SCOP label is checked to determine whether domains in the pair are actual in the same SCOP class. If both domains in the pair have the same SCOP label, they are considered as homologous domains. Using the information in the ordered list, we measure the coverage and the specificity for various k cutoffs, where k is the top k high scoring (or low distance) pairs in the list. The coverage(k) and specificity(k) are defined as:

$$\begin{aligned} Coverage(k) &= N^{homologs}(k)/N^{homologs}(Q*N) \\ Specificity(k) &= N^{homologs}(k)/k \end{aligned}$$

where Q is the number of query domains and N is the number of target domains in database, $N^{homologs}(Q*N)$ is the total number of homologous domain pairs found in the entire query and target domains pairs, and $N^{homologs}(k)$ is the number of homologous domain pairs detected in the top k pairs. ROC curves produced using this method are plotted, and are shown in Figure 1, 2, and 3 for the SCOP family, superfamily, and fold levels respectively. The results in these Figures show that proCC is more effective than SGM for detecting homologous domain pairs at the SCOP family, superfamily, and fold levels.

[1] Wallqvist A. *et al.* Iterative sequence/secondary structure search for protein homologs: comparison with amino acid sequence alignments and application to fold recognition in genome databases, Bioinformatics 2002 16(11):988-1002

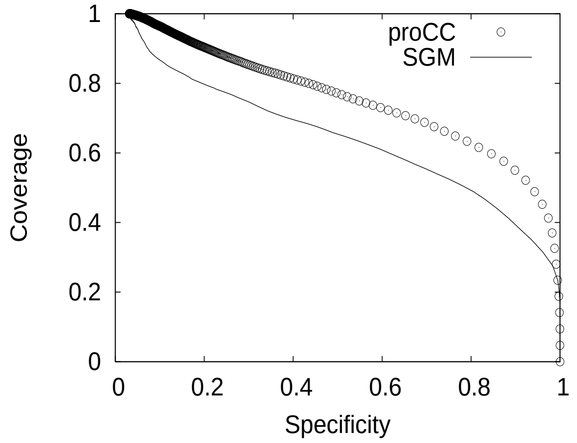

Figure 1: ROC curve at the SCOP family level

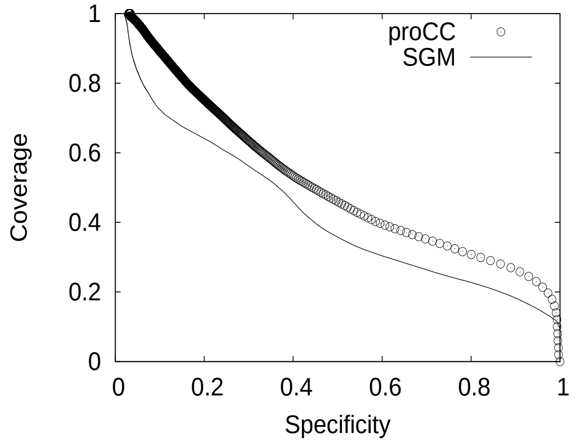

Figure 2: ROC curve at the SCOP superfamily level

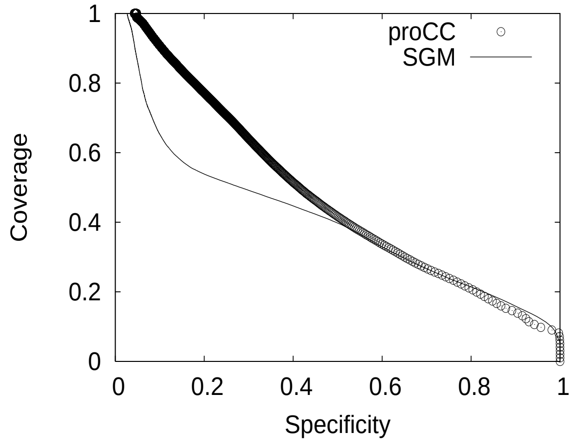

Figure 3: ROC curve at the SCOP fold level
